# The Clinical Impact of Accurate Cystine Calculi Characterization Using Dual-Energy Computed Tomography

**DOI:** 10.1155/2015/801021

**Published:** 2015-11-25

**Authors:** William E. Haley, El-Sayed H. Ibrahim, Mingliang Qu, Joseph G. Cernigliaro, David S. Goldfarb, Cynthia H. McCollough

**Affiliations:** ^1^Mayo Clinic, Jacksonville, FL 32224, USA; ^2^University of Michigan, Ann Arbor, MI 48109, USA; ^3^Mayo Clinic, Rochester, MN 55905, USA; ^4^New York Harbor VA Healthcare System, Brooklyn, NY 11209, USA

## Abstract

Dual-energy computed tomography (DECT) has recently been suggested as the imaging modality of choice for kidney stones due to its ability to provide information on stone composition. Standard postprocessing of the dual-energy images accurately identifies uric acid stones, but not other types. Cystine stones can be identified from DECT images when analyzed with advanced postprocessing. This case report describes clinical implications of accurate diagnosis of cystine stones using DECT.

## 1. Introduction

Cystinuria is the most common of the inherited kidney stone diseases, accounting for about 1-2% and 25% of adult and pediatric patients, respectively, with kidney stones [1]. It is an autosomal recessive disorder caused by mutations in the SLC3A1 and SLC7A9 genes, leading to an increased excretion of the amino acid cystine, which is poorly soluble in urine, resulting in the formation of recurrent kidney stones [2]. The divergent treatment strategies for cystine and calcium stones reflect the importance of identifying the stone composition accurately [3]. Dual-energy computed tomography (DECT) is a relatively new imaging modality that has proven successful in differentiating between uric acid (UA) and non-UA stones with near 100% specificity for stones greater than 3 mm in size [4, 5]. In contrast to conventional CT, DECT utilizes X-ray tube and detector structures, which are set at different tube potentials, to simultaneously acquire two data sets that allow differentiation of stone materials based on the attenuation ratio between the two peak X-ray energies [5, 6]. This capability potentially obviates the need for stone analysis to guide treatment. Nevertheless, with the current standard processing algorithm of DECT images, all non-UA stones are characterized as a single group without being further separated and are not differentiated from the more common calcium stones and other less common types including cystine [5]. We report a case of a patient with a long history of presumed calcium stones, in whom advanced processing of the DECT images correctly identified cystine stones, leading to change in treatment and resulting in improvement in stone-related outcomes.

## 2. Case Presentation

A 65-year-old man presented for evaluation with recent onset of intermittent left and right flank pain and nausea. History was notable for numerous stones over 35 years often requiring urological procedures, including extracorporeal shock wave lithotripsy (ESWL), ureteroscopy, and percutaneous nephrolithotomy. Increasing stone burden was documented on serial X-ray studies. Other medical histories included pulmonary sarcoidosis, with no recent hypercalcemia. The patient had no family history of kidney stones. Body mass index was 28 kg/m^2^; physical examination was unremarkable. Laboratory testing revealed normal serum creatinine, calcium, phosphorus, and electrolytes. Urinalysis showed 21 WBC/hpf, 6 RBC/hpf, and pH 6. 24 h urine panel included volume 2750 mL, calcium 206 mg/24 h, sodium 336 mEq/24 h, citrate 976 mg/24 h, uric acid 853 mg/24 h, oxalate 44 mg/24 h, phosphorus 1568 mg/24 h, and pH 6.2, with elevated supersaturation of calcium phosphate (brushite and apatite).

**Figure 1:**
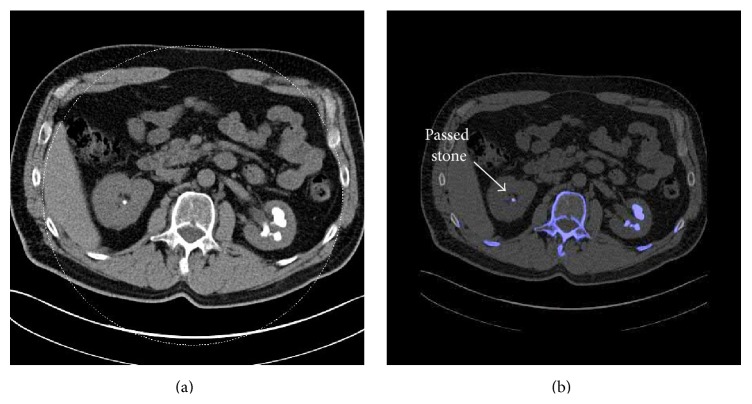
Conventional CT (a) and DECT (b) images showing a large staghorn stone and a small stone in the left and right kidneys, respectively. The standard DECT postprocessing algorithm colors all non-uric-acid stones blue. A follow-up scan confirmed the location of the passed stone.

**Figure 2:**
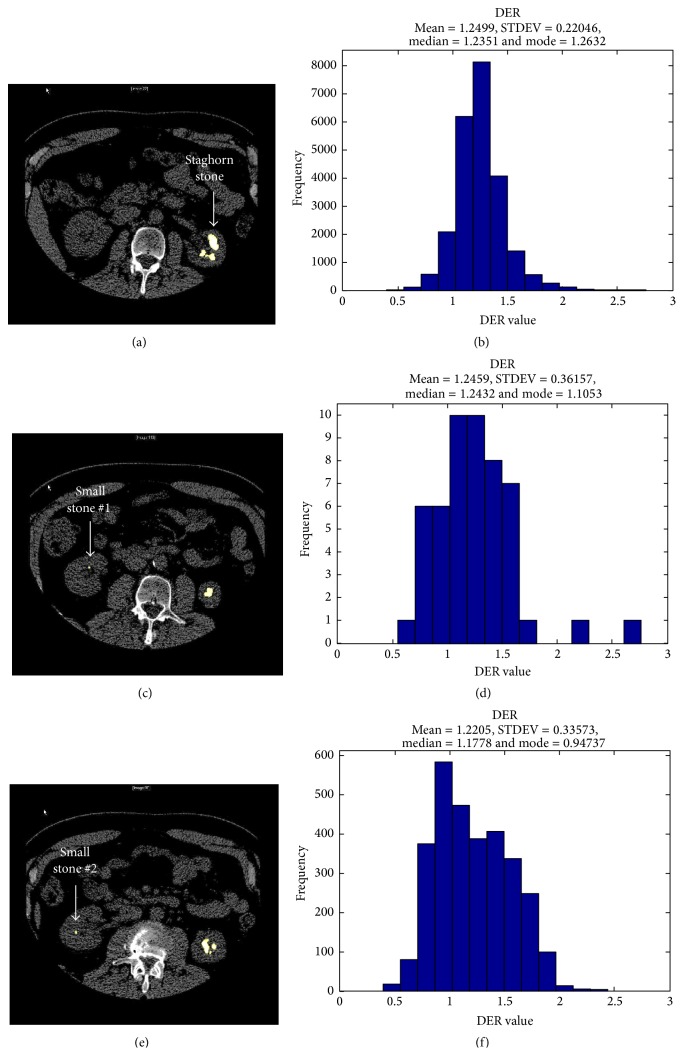
DECT scan with advanced postprocessing, which identified the stone material as cystine, in (a) left-side staghorn stone appears in this slice, and in (b), (c) two small right-side stones appear in these slices labeled small stone #1 and small stone #2, assigned a unique color (yellow). Histograms of the dual-energy ratio (DER) distribution showing mean ± SD = 1.25 ± 0.22 of staghorn stone (d), small stone #1 in (e) = 1.24 ± 0.36, and small stone #2 in (f) = 1.22 ± 0.34, which are in the range associated with cystine.

A DECT scan was performed using a Somatom Definition Flash dual-source dual-energy CT scanner (Siemens Healthcare, Forchheim, Germany), which utilizes 2 independent X-ray tube and detector structures and includes a tin filter for better separation between different X-ray beam spectra [7]. A dedicated renal stone imaging protocol was used, without intravenous or oral contrast. The DECT peak tube potentials (kVp) were set to 80 kV and 140 kV. Images were obtained helically from the diaphragm to the pubic symphysis with 0.75 mm slice thickness. Data were reconstructed on a multimodality workstation (Syngo Kidney Stone, Siemens Healthcare, Forchheim, Germany), which color-codes the UA and non-UA stones in red and blue, respectively, based on a 3-material decomposition algorithm (water, calcium, and uric acid). Images were reconstructed with a 0.75 mm slice thickness and 0.5 mm interval using a D30f convolution kernel for enhanced resolution. These images revealed bilateral renal calculi: a large staghorn on the left and two smaller stones on the right. There were no obstructing stones. All stones were characterized as non-UA (color-coded in blue), as shown in Figure 1. Images were postprocessed using an advanced algorithm previously demonstrated to differentiate different types of non-UA stones. The postprocessing involved manually determining a region of interest, followed by software segmentation from surrounding tissues using a predefined CT number threshold and extraction of a 3D volume of interest containing the stone. The CT number was calculated at 80 kV and divided by the number at 140 kV for each voxel and the average dual-energy ratio (DER) value of all voxels within the segmented stone was used for characterizing the composition [8, 9]. The DER measurements were calculated for the large left kidney stone (DER (mean ± SD) = 1.25 ± 0.22), and the right-side stones (stone #1 DER = 1.24 ± 0.36 and stone #2 DER = 1.22 ± 0.34) (Figure 2). These DER values lie in the range associated with cystine [8, 9].

Analysis performed on a spontaneously passed stone by infrared spectroscopy revealed 100% cystine. Cystine excretion in 24 h urine was measured: 3.5 mmol (875 mg)/24 h. The patient was treated with tiopronin and potassium citrate. He was instructed to increase fluid intake, decrease dietary sodium, and moderate protein intake. On this regimen, follow-up for 2 years has been remarkable for stability of residual stones with no new stone growth and no urological procedures required.

## 3. Discussion

In this case report we demonstrate that cystine stones can be accurately identified from DECT images when analyzed with advanced postprocessing, and we describe clinical implications including significant impact on prescribing appropriate treatment that led to improved outcomes. This observation has significant clinical impact because the medical treatment of cystine stones requires a different approach from calcium stones [3]. Indeed, the prescribed regimen aimed at prevention of cystine stone growth proved to be highly successful in this case. Prior to that, the patient had suffered from a long history of presumed calcium-based stones, including frequent attacks and urological procedures, including ESWL, ureteroscopy, and percutaneous nephrolithotomy. This patient's history of sarcoidosis, with its association with calcium stones, clouded the clinical picture. On presentation, he was found to have increasing stone burden on serial X-ray studies including the development of a symptomatic staghorn calculus.

DECT has been successfully used in recent years to distinguish UA from non-UA stones [5]. It allows for immediate identification of UA stones, raising the prospect of medical dissolution as an option, in addition to or in place of urological procedures. Whereas non-UA stones comprise about 90% of stones [5], the standard commercially available DECT 3-material decomposition image processing algorithm cannot differentiate between different non-UA stone types with sufficient reliability. Consequently, all non-UA stones are grouped together and assigned the same color. Distinguishing the more common non-UA stones, calcium oxalate and calcium phosphate, from less common stone types including cystine is important to assure the best outcome, as illustrated in this case report. Cystine stones, like uric acid stones, are often less radiopaque than calcium stones and may not be seen on plain films of the abdomen. Cystine stones are detectable but indistinguishable from other composition types on conventional noncontrast CT as well as standard DECT (Figure 1).

Although cystinuria meets the criteria of a rare disease (less than 200,00 cases in the United States), it is not as uncommon as one might expect from that designation, given that kidney stones affect about 10% of the adult US population and 1-2% of those may have cystinuria; the true prevalence may be even higher as some proportion of affected persons never have the diagnosis made. The present case highlights the potential for misdiagnosis of rare stones and may help explain the discrepancy in case-finding compared to estimated prevalence noted by the Rare Kidney Stone Consortium (http://www.rarekidneystones.org/). A number of factors contribute to the underrecognition of this disease. The presence of pathognomonic hexagonal cystine crystals on urinalysis is noted in only a fraction of cases. The diagnosis of cystinuria may be made based on measuring 24 h urine cystine excretion; however, this can be problematic [2, 10]. Nitroprusside screening of urine, performed by many laboratories specializing in assessment of urinary risk factors, is inexpensive and sensitive, but many patients never have urine sent to such specialty labs. Stone analysis by infrared spectroscopy or X-ray diffraction confirming cystine is diagnostic; however, such analysis is often not available to assist with diagnosis. Family history is usually not helpful, unless siblings are known to be affected. Absent the latter, a nitroprusside screening test, or the pathognomonic crystals on urinalysis, there would be little suspicion for this diagnosis.

The natural history of cystine stone disease is variably expressed. Most affected individuals present in childhood and 75% by teen years marked by multiple stone recurrences; however, a surprising number manifest in 40–80 year olds [11]. A significant proportion of adults develop renal pathological changes and loss of kidney function relating to crystalline nephropathy, in addition to recurrent obstructive uropathy and repeated urological interventions [2, 12]. Therefore, making the correct diagnosis has great clinical importance, reducing urological procedures and potential complications through medical management. Hydration, dietary modification, and urinary alkalinization have been successfully used for prevention of new cystine stones [3]. The restriction of dietary protein and sodium reduces urinary excretion of cystine, alkalinizing the urine improves cystine solubility, and cystine-binding thiol drugs, tiopronin or d-penicillamine, form soluble drug-cysteine complexes [10].

CT radiation dose deserves comment in the context of this report. Thomas reported a mean effective dose 2.7 mSv among 40 patients using a similar low-dose unenhanced DECT protocol, equipment, and settings [8]. With this technology, radiation dose is increased in obese patients. 100 kV can be used for the low energy setting to enhance image quality [5]. Experts have urged caution in the use of effective dose in estimating risks of ionizing radiation, especially in diagnostic X-ray studies. Low dose stone studies fall within the range of natural background levels of radiation found in our environment and any adverse health effect at such low dose levels of radiation used in medical imaging is either too small to be demonstrated or is nonexistent [13, 14].

Important questions remain. The sensitivity and specificity of DECT for the diagnosis of cystine calculi need further study. The detection limit of DECT technology is not known. 3D imaging may be utilized to quantify cystine burden and track response to treatment. Mixed stones are common; to what extent DECT with its ability to separate 5 types of kidney stones (uric acid, cystine, struvite, calcium oxalate, and calcium phosphate) [5, 8] fits into the management of patients in general practice is yet to be established.
